# Evidence for the role of Irk2 and Irk5 in ATP and metabolism regulation in *Cryptococcus neoformans*

**DOI:** 10.3389/fcimb.2025.1600041

**Published:** 2025-06-18

**Authors:** Yuanyuan Ma, Jianhua Qu, Mingming Xu, Nuoya Zhou, Xiaoya Chen, Yu Han, Peng Xue

**Affiliations:** ^1^ Nantong Key Laboratory of Environmental Toxicology, Department of Occupational Medicine and Environmental Toxicology, School of Public Health, Nantong University, Nantong, China; ^2^ Wisdom Lake Academy of Pharmacy, Xi’an Jiaotong-Liverpool University, Suzhou, China

**Keywords:** cryptococcosis, Irk2, Irk5, ATP, proteome, metabolomics

## Abstract

**Introduction:**

*Cryptococcus neoformans*, a human fungal pathogen, harbors the kinases Irk2 and Irk5, which are classified within the APH phosphotransferase, AGC/YANK protein kinase, and diacylglycerol kinase-like kinase families. Both Irk2 and Irk5 are pivotal for virulence during lung and brain infections. Previous studies have demonstrated that deletion of the *IRK5* gene results in a significant reduction in cell wall associated melanin production, a vital virulence factor that facilitates evasion of host immune responses, while deletion of *IRK2* does not manifest any notable phenotypic alterations.

**Methods:**

To investigate the impact of *IRK2* or *IRK5* deletion, we generated targeted deletion mutants for each gene. Following the creation of these mutants, we conducted mass spectrometry analyses to evaluate changes in their proteomic and metabolomic profiles. Moreover, we measured intracellular ATP levels in both the wild-type and mutant strains to assess modifications in ATP synthesis.

**Results:**

Mass spectrometry analyses revealed significant alterations in protein and metabolite expression levels in the *IRK2* or *IRK5* deletion mutant compared to the wild-type strain. Furthermore, the deletion of either *IRK2* or *IRK5* resulted in notable changes in intracellular ATP levels.

**Conclusion:**

This study suggests that the core virulence kinases Irk2 and Irk5 may play roles in regulating ATP levels and metabolic pathways. By elucidating the effects of these kinases on the proteomic and metabolomic profiles of *C. neoformans*, this research contributes to our understanding of the underlying molecular mechanisms involved in the pathogenesis of this pathogen.

## 1 Introduction

*Cryptococcus neoformans* is a prominent encapsulated yeast and a significant fungal pathogen, particularly affecting immunocompromised individuals, such as those living with HIV/AIDS (Buchanan and Murphy, 1998; Kronstad et al., 2011; Iyer et al., 2021). This fungus is responsible for cryptococcal meningoencephalitis, leading to approximately 200,000 deaths annually in the HIV/AIDS population (Park et al., 2009; Rajasingham et al., 2017; Mourad and Perfect, 2018; Shroufi et al., 2021; Denning, 2024). Notably, it is one of four species identified by the World Health Organization as a critical priority among human pathogenic fungi (WHO, 2022). Infections caused by *C. neoformans* primarily target the lungs and central nervous system, resulting in considerable morbidity and mortality (Zhou et al., 2024). A comprehensive understanding of the virulence mechanisms employed by *C. neoformans* is essential for developing effective therapies and preventive strategies against cryptococcosis, a major global health concern. The virulence of this fungus is multifactorial, with key factors including its thick polysaccharide capsule, melanin production, and remarkable adaptability to diverse host environments (Garcia-Rubio et al., 2019; Xue et al., 2023). The capsule is essential for evading the host immune response, while melanin enhances resistance to oxidative stress and phagocytosis. Additionally, *C. neoformans* thrives at elevated temperatures and can utilize various carbon sources, further enhancing its survival and pathogenicity within the host (Berguson et al., 2022; He et al., 2024).

In *C. neoformans*, 34 kinases have been identified as core virulence kinases, essential for infections in both the lungs and brain, including Irk2 and Irk5 (Jin and Lee, 2020; Lee and Hong, 2020). Some of these kinases serve as signaling components within established pathways, including the cAMP signaling cascade, the high osmolarity glycerol (HOG) response pathway, and the cell wall integrity MAPK pathway (Lee and Hong, 2020). However, the regulatory mechanisms of the kinases Irk2 and Irk5 remain unclear. Irk2 and Irk5 belong to distinct families of protein kinases, such as APH phosphotransferases and AGC/YANK protein kinases, alongside diacylglycerol kinase-like kinases. Irk5 plays a significant role in melanin production; its deletion results in a substantial reduction in melanin synthesis, potentially compromising *C. neoformans* ability to resist host immune responses (Lee and Hong, 2020). Conversely, the deletion of *IRK2* does not lead to significant phenotypic changes *in vitro* (Lee and Hong, 2020), warranting further investigation into its role in virulence and cellular metabolism. This study aims to investigate the roles of both Irk2 and Irk5 in *C. neoformans* by examining proteomic and metabolomic changes following gene deletion. Utilizing quantitative proteomics and untargeted metabolomics, we aim to identify shifts in protein expression and metabolite levels. Preliminary findings suggest that both Irk2 and Irk5 may regulate the levels of proteins and metabolites associated with mitochondrial function, which is critical for ATP production. Furthermore, the deletion of these kinases led to significant changes in ATP levels, emphasizing their involvement in cellular metabolism, particularly mitochondrial function. This research enhances our understanding of the roles of Irk2 and Irk5 in cellular metabolism.

## 2 Materials and methods

### 2.1 Strains, growth conditions, and measurement of intracellular ATP levels

This study employed the wild-type (WT) strain of *Cryptococcus neoformans* var. *grubii* H99, as well as the independent deletion mutants *irk2*Δ and *irk5*Δ. The primers used for the generation of these mutants are detailed in **Supplementary Table S1**. The gene sequence encoding the Irk2 homolog (CNAG_02542) was obtained from the *C. neoformans* var. *grubii* serotype A genome database (https://www.broadinstitute.org/fungal-genome-initiative/cryptococcus-neoformansserotype-genome-project). To construct the *irk2*Δ mutant, we performed homologous recombination to replace the genetic locus containing the 795-base pair (bp) open reading frame of the *IRK2* gene, which has a total coding region of 912-bp, with a gene-specific deletion cassette. This cassette was amplified using the primers Irk2-UP-F, Irk2-UP-R, neoF, neoR, Irk2-Down-F, and Irk2-Down-R, and was subsequently introduced into the WT strain via biolistic transformation, following established protocols (Yu et al., 2004; Kim et al., 2009; Brand and MacCallum, 2012). Verification of positive transformants was conducted using polymerase chain reaction (PCR). In a parallel approach, the gene sequence for the Irk5 homolog (CNAG_03811) was also sourced from the *C. neoformans* var. *grubii* serotype A genome database (https://www.broadinstitute.org/fungal-genome-initiative/cryptococcus-neoformansserotype-genome-project).The *irk5*Δ mutant was generated by substituting the genetic locus containing the 1367-bp open reading frame of the *IRK5* gene, which has a total coding region of 1455-bp, with a gene-specific deletion cassette using the primers Irk5-UP-F, Irk5-UP-R, neoF, neoR, Irk5-Down-F, and Irk5-Down-R. This deletion cassette was introduced into the WT strain through biolistic transformation, adhering to previously established methodologies (Yu et al., 2004; Kim et al., 2009; Brand and MacCallum, 2012), and PCR was utilized for confirmation of successful transformants (**Supplementary Figure S1**). To evaluate differences in intracellular ATP levels, proteomics, and metabolomics between the WT and mutant strains, cells were cultured overnight in YPD medium at 30°C with shaking at 150 rpm. ATP quantification was performed using the BacTiter-Glo Microbial Cell Viability Assay kit (Promega, USA), with measurements taken using a microplate reader (TECAN Infinite E Plex). For subsequent proteomics and metabolomics analyses, cells were harvested after 16 hours of shaking at 150 rpm and 30°C. The cells were then washed twice with distilled water (dH_2_O), and the resulting pellets were frozen in liquid nitrogen prior to storage at -80°C.

### 2.2 Proteome studies

The initial phase of the proteomic analysis involved extracting proteins from snap-frozen biological samples of cells grown in YPD media during the logarithmic phase. Fungal cells were first ground in liquid nitrogen, followed by sonication in a lysis buffer containing 10 mM dithiothreitol, 1% Triton X-100, 1% protease inhibitor cocktail, 3 μM TSA, 50 μM PR-619, 2 mM EDTA, 50 mM NAM, and 1% phosphatase inhibitor to prevent phosphorylation. Equal volumes of Tris-saturated phenol (pH 8.0) were mixed in, and the solution was vortexed for 5 minutes. The mixture was then centrifuged at 4°C for 10 minutes at 5,500g, and the upper phenol layer was carefully transferred to a new centrifuge tube. To precipitate the proteins, a minimum of four volumes of methanol saturated with ammonium sulfate were added, and the mixture was kept at -20°C for at least 6 hours. After another centrifugation at 4°C for 10 minutes, the supernatant was removed. The resulting protein pellet was washed three times, first with ice-cold methanol and then with ice-cold acetone. Finally, the proteins were dissolved in 8 M urea, and their concentration was measured using the BCA assay according to the manufacturer’s guidelines. Once the protein concentration was quantified, trypsin digestion was initiated by precipitating the proteins with 20% (m/v) trichloroacetic acid. The precipitated proteins underwent additional washes and were redissolved in 200 mM triethylammonium bicarbonate buffer. For the initial digestion, trypsin was added at a 1:50 mass ratio relative to the protein and allowed to digest overnight. Afterward, the sample was reduced with 5 mM dithiothreitol for 30 minutes at 56°C, followed by alkylation with 11 mM iodoacetamide in the dark at room temperature for 15 minutes. The resulting peptides were desalted using a Strata X solid-phase extraction column. For liquid chromatography-tandem mass spectrometry (LC-MS/MS) analysis, the tryptic peptides were dissolved in solvent A and loaded onto a custom-made reversed-phase analytical column (25 cm length, 100 μm inner diameter). The mobile phase consisted of two solvents: solvent A contained 0.1% formic acid in 2% acetonitrile in water, while solvent B contained 0.1% formic acid in acetonitrile. Peptide separation was performed using a carefully calibrated gradient: from 0 to 14 minutes, the solvent B concentration increased from 6% to 24%; from 14 to 16 minutes, it rose from 24% to 35%; from 16 to 18 minutes, it escalated from 35% to 90%; and from 18 to 20 minutes, it was held at 90% B. The flow rate was maintained at a constant 500 nl/min on the Easy-nLC1000 UHPLC system (Bruker Daltonics). The separated peptides were then introduced into a capillary source for analysis on the timsTOF Pro mass spectrometer, where an electrospray voltage of 1.75 kV was applied to promote peptide ionization. The analysis captured precursor ions and their fragments in the TOF detector, with the timsTOF Pro operating in data-independent parallel accumulation serial fragmentation (dia-PASEF) mode. The full MS scan range was set from 300 to 1500 m/z, allowing broad detection of ionized peptides, while 20 PASEF-MS/MS scans were collected per cycle. The MS/MS scans were restricted to a range of 400 to 850 m/z, with an isolation window of 7 m/z. The DIA data obtained were analyzed with the DIA-NN search engine (version 1.8) (Demichev et al., 2020, 2022). Tandem mass spectra were matched against the database of *C. neoformans* var. *grubii* serotype A strain H99 (*C. neoformans* var. *grubii* serotype A strain H99–235443 PR 20240409.fasta, containing 7429 entries), concatenated with a reverse decoy database to enhance result reliability. Trypsin/P was specified as the cleavage enzyme, allowing for a maximum of one missed cleavage. Fixed modifications included the excision of N-terminal methionine and carbamidomethylation of cysteine residues, with a false discovery rate (FDR) maintained below 1% to ensure high confidence in protein identifications. Bioinformatics analyses were essential to this study, using Gene Ontology (GO) to annotate proteins by their cellular components, molecular functions, and biological processes. The GO annotation process involved utilizing eggnog-mapper software to obtain GO IDs from identified proteins in the EggNOG database, followed by functional classification analysis of these proteins (Cantalapiedra et al., 2021). The KEGG (Kyoto Encyclopedia of Genes and Genomes) pathway annotations established connections between identified proteins and recognized biological pathways, providing important insights into their functional roles. To annotate protein pathways, the KEGG pathway database was utilized, and proteins were identified via BLAST comparisons (blastp, evalue ≤ 1e-4) (Kanehisa and Goto, 2000; Kanehisa et al., 2016). For each sequence, the annotation was based on the comparison result with the highest score. Specialized software tools predicted the subcellular localization of eukaryotic proteins, while transcription factors were identified through established databases. Functional enrichment analysis employed Fisher’s exact test, concentrating on differentially expressed proteins with the identified proteins serving as a reference background. Significant functional terms were defined by a fold enrichment greater than 1.5 and a p-value below 0.05. Lastly, enrichment-based clustering was executed for data visualization via hierarchical clustering, offering a comprehensive overview of functional classifications and their significance within the analyzed proteomes. The procedure was repeated with three independent biological replicates for each strain, including mutants and WT.

### 2.3 Metabolomics studies

Sample preparation for metabolomics studies began with the thawing of snap-frozen biological samples of cells cultivated in YPD media and collected during the logarithmic phase. These samples, stored at -80°C, were thawed on ice to preserve stability and prevent degradation. They were homogenized for 20 seconds using a grinder and then mixed with 400 μL of a methanol-water solution (4:1, V/V) containing an internal standard. The mixture was vortexed for 3 minutes, followed by three freeze-thaw cycles alternating between liquid nitrogen and dry ice, with additional vortexing after each cycle. Centrifugation at 12,000 rpm for 10 minutes at 4°C allowed for the collection of 300 μL of the supernatant, which was stored at -20°C for 30 minutes before a second centrifugation step. A 200 μL aliquot of the supernatant was prepared for subsequent LC-MS analysis. All samples underwent two distinct LC-MS methodologies. One aliquot was analyzed under positive ionization conditions using a T3 column (Waters ACQUITY Premier HSS T3 Column, 1.8 µm, 2.1 mm × 100 mm). The mobile phase comprised 0.1% formic acid in water (solvent A) and 0.1% formic acid in acetonitrile (solvent B), following a gradient schedule: increasing from 5% to 20% B over 2 minutes, escalating to 60% B over the next 3 minutes, further increasing to 99% B within 1 minute, maintaining this for 1.5 minutes, and then returning to 5% B within 0.1 minutes, followed by an additional hold for 2.4 minutes. The analytical conditions included a column temperature of 40°C, a flow rate of 0.4 mL/min, and an injection volume of 4 μL. A second aliquot was evaluated under negative ionization conditions, utilizing the same elution gradient as for the positive mode. Data acquisition was performed in information-dependent acquisition (IDA) mode using Analyst TF 1.7.1 Software (Sciex, Concord, ON, Canada). The ion source parameters were configured as follows: ion source gas 1 (GAS1) at 50 psi; ion source gas 2 (GAS2) at 50 psi; curtain gas (CUR) at 25 psi; temperature (TEM) set to 550°C; declustering potential (DP) at 60 V for positive mode and -60 V for negative mode; and ion spray voltage floating (ISVF) at 5000 V for positive and -4000 V for negative ionization conditions. The Time-of-Flight (TOF) MS scan parameters were set with a mass range of 50–1000 Da, an accumulation time of 200 ms, and dynamic background subtraction activated. For product ion scans, parameters included a mass range of 25–1000 Da, an accumulation time of 40 ms, collision energies of 30 V for positive and -30 V for negative modes, a collision energy spread of 15, a resolution setting of UNIT, charge state limited to 1, intensity set at 100 counts per second (cps), exclusion of isotopes within 4 Da, a mass tolerance of 50 ppm, and a maximum of 18 candidate ions monitored per cycle. Differential metabolites were identified based on Variable Importance in Projection (VIP) values (VIP > 1) and *P* values (*P* < 0.05) using Orthogonal Partial Least Squares Discriminant Analysis (OPLS-DA). Identified metabolites were annotated through the KEGG Compound database (http://www.kegg.jp/kegg/compound/) and subsequently linked to the KEGG Pathway database (http://www.kegg.jp/kegg/pathway.html). The pathways with significant enrichment were determined using a hypergeometric test, calculating the P-value based on the provided list of metabolites. Three independent biological replicates were performed for each strain, including both mutants and WT.

## 3 Results

### 3.1 Proteome and metabolomics analyses of *irk2*Δ mutant

**Figure 1:**
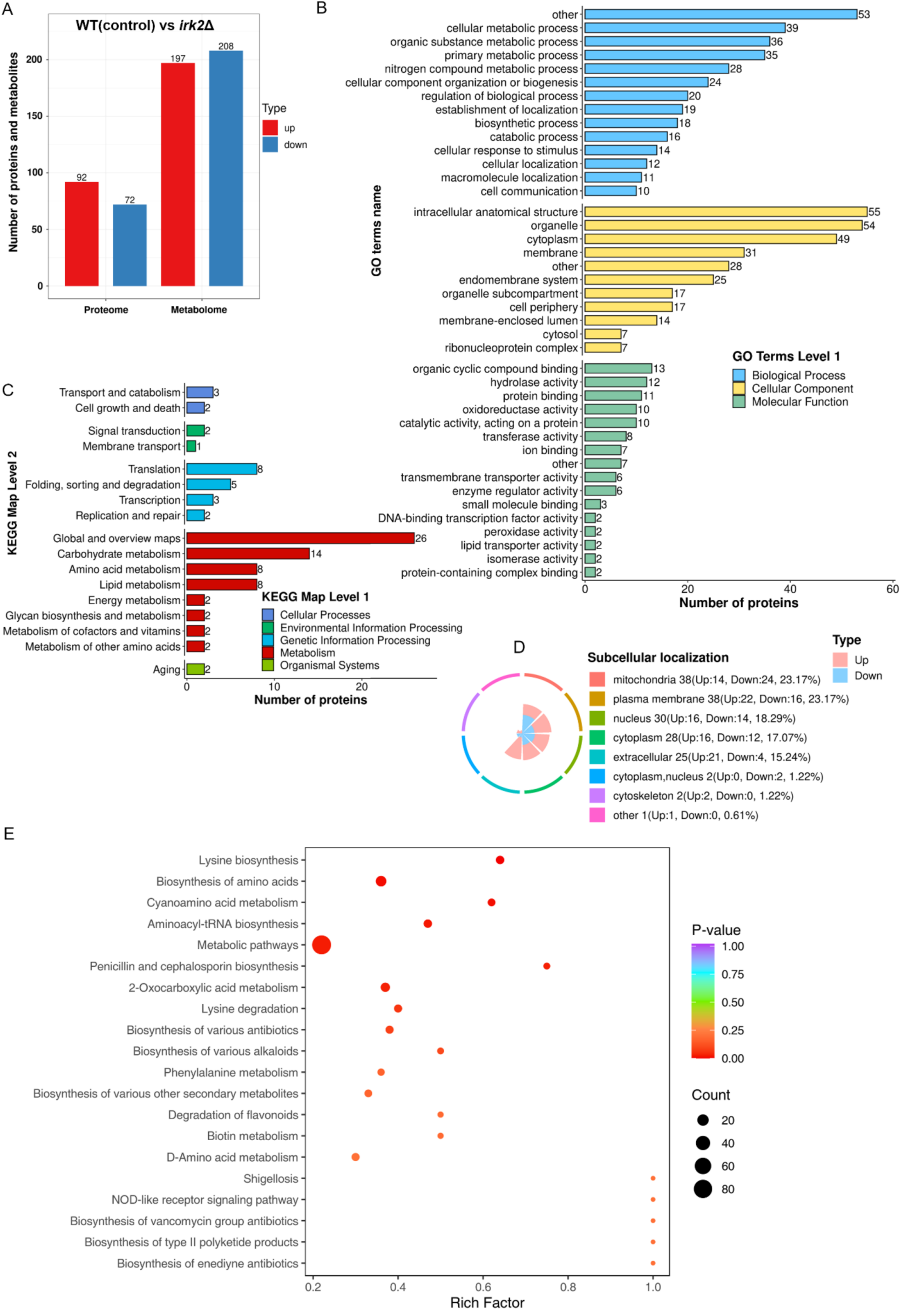
*IRK2* gene knockout resulted in alterations to the protein and metabolic profiles of *C*. *neoformans*. **(A)** Bar chart depicting the number of differentially expressed proteins and metabolites. For the proteome, proteins were considered significantly up-regulated (red) if the ratio (experimental group/control group) > 1.5 with t-test probabilities < 0.05, and significantly down-regulated (blue) if the ratio < 0.67 with t-test probabilities < 0.05. For the metabolomics, metabolites were deemed significantly up-regulated (red) or down-regulated (blue) based on t-test probabilities < 0.05. **(B)** GO classification of regulated proteins. This represents a functional enrichment cluster analysis of differentially expressed proteins. **(C)** KEGG pathway classification of regulated proteins, also indicating functional enrichment cluster analysis. **(D)** Subcellular localization of differentially expressed proteins. **(E)** KEGG enrichment analysis of differentially expressed metabolites.

The Irk2 kinase is crucial for the virulence of *C. neoformans* in lung and brain infections. However, the absence of the *IRK2* gene does not induce significant phenotypic changes (Lee and Hong, 2020). This investigation delves into the impact of *IRK2* deletion on the proteomic and metabolomic profiles of *C. neoformans*. Utilizing data-independent acquisition (DIA)-based quantitative proteomics, we compared the proteomic profiles of WT and *irk2*Δ mutant strains, successfully identifying differentially expressed proteins. Additionally, we conducted untargeted metabolomics analyses to assess alterations in metabolite levels associated with the deletion. Our proteomic analysis successfully identified a total of 4931 proteins from the 7429 present in *C. neoformans*. Significant changes in protein expression were defined by expression ratios falling below 0.67 or exceeding 1.5, with a significance threshold established at t-test probabilities of less than 0.05. These thresholds are widely accepted methods in proteomic studies and have been utilized in previous research to ensure robust and meaningful results. Additionally, we performed multiple test corrections for our t-tests to ensure the reliability of our results. An overview of these differences is depicted in **Supplementary Table S2**. The deletion of *IRK2* resulted in the differential expression of proteins corresponding to 164 genes, inclusive of 92 upregulated and 72 downregulated proteins (**Figure 1A**). GO analysis revealed that these regulated proteins are involved in various biological processes, such as cellular metabolic processes, organic substance metabolism, primary metabolic activities, and nitrogen compound metabolism. The proteins were further categorized based on their cellular components and molecular functions (**Figure 1B**; **Supplementary Figure S2**). KEGG pathway analysis indicated that the regulated proteins are implicated in several metabolic pathways, including carbohydrate metabolism, amino acid metabolism, lipid metabolism, energy metabolism, glycan biosynthesis, and the metabolism of cofactors and vitamin (**Figure 1C**). The subcellular localization of the differentially expressed proteins, encompassing those associated with mitochondria, plasma membranes, nuclei, cytoplasm, extracellular spaces, and cytoskeletons, is illustrated in **Figure 1D**. Furthermore, in the metabolomics aspect of our study, **Figure 1A** summarizes the observed changes in metabolite levels. The deletion of *IRK2* produced distinct metabolic profiles characterized by 405 individual alterations, with 197 metabolites exhibiting increased levels and 208 displaying decreased levels. The KEGG enrichment analysis of the differentially expressed metabolites is illustrated in **Figure 1E**; **Supplementary Figure S3**. Notable pathways affected include aminoacyl-tRNA biosynthesis, biosynthesis of amino acids, and metabolic pathways (**Figure 1E**; **Supplementary Figure S3**). Overall, the knockout of *IRK2* significantly influences both protein expression and metabolite profiles in *C. neoformans*.

### 3.2 Proteome and metabolomics analyses of *irk5*Δ mutant

**Figure 2:**
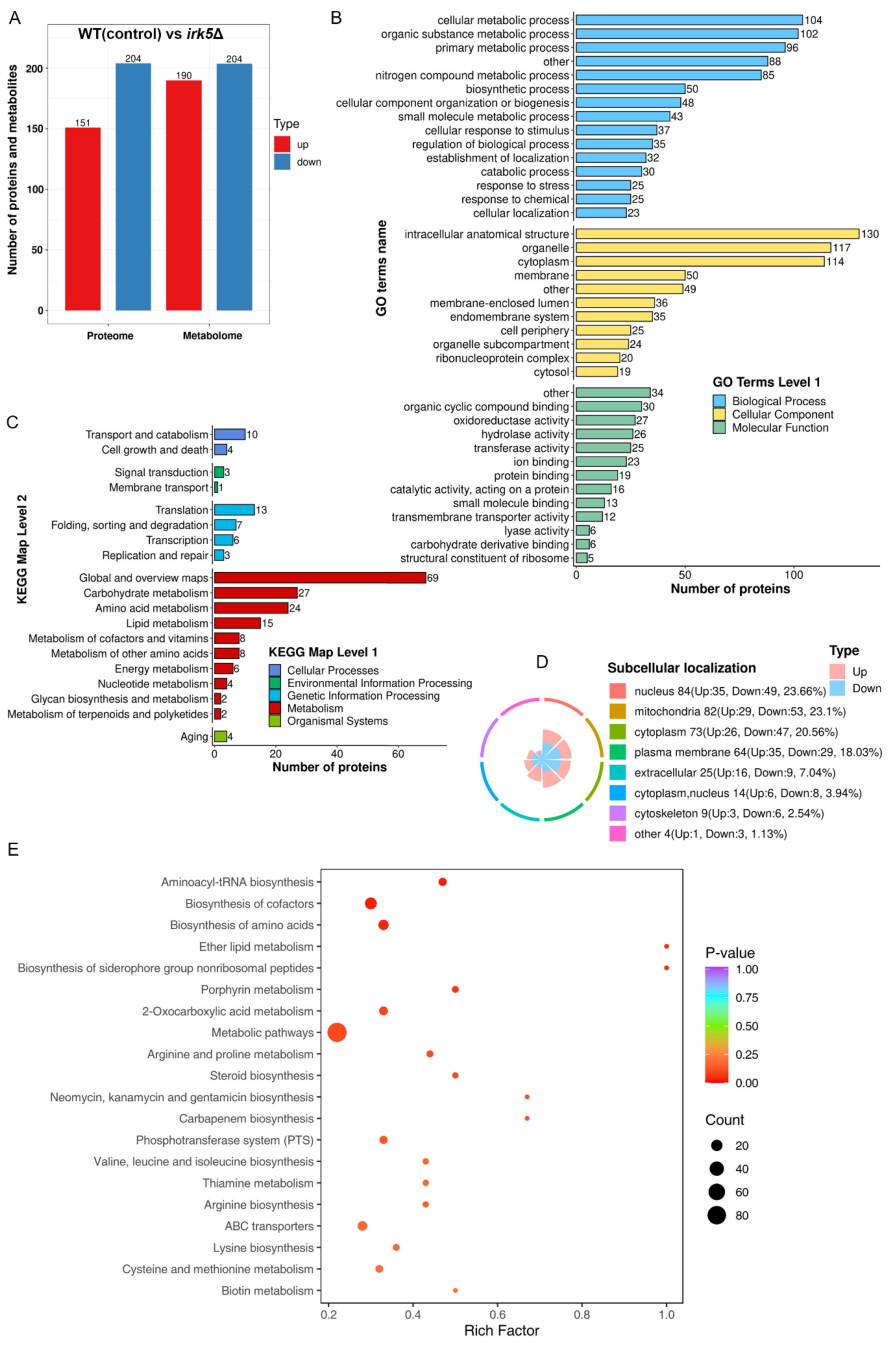
Knockout of *IRK5* gene modified the protein and metabolite profiles in *C*. *neoformans*. **(A)** Bar chart showing differentially expressed proteins and metabolites: up-regulated proteins (red) with a ratio > 1.5 and *p* < 0.05; down-regulated proteins (blue) with a ratio < 0.67 and *p* < 0.05; metabolites with *p* < 0.05. **(B)** GO classification of differentially expressed proteins based on functional enrichment analysis. **(C)** KEGG pathway analysis of regulated proteins. **(D)** Localization of various proteins with differential expression at the subcellular level. **(E)** KEGG analysis for enrichment of metabolites that are differentially expressed.

Irk5, like Irk2, participates in critical signaling pathways associated with virulence of *C. neoformans* during infections in both the lungs and the brain (Lee and Hong, 2020). To further elucidate the regulatory functions of these kinases, we performed comprehensive proteomic and metabolomic analyses on the *irk5*Δ mutant. Our proteomic analysis successfully identified 4931 proteins from the total of 7429 present in *C. neoformans*. Significant alterations in protein expression were similarly defined as ratios below 0.67 or above 1.5, with a significance threshold set at t-test probabilities of less than 0.05. To ensure the reliability of our results, we also applied multiple test corrections to our t-tests. An overview of the observed differences is presented in **Supplementary Table S2**. The deletion of *IRK5* led to the differential expression of proteins in 355 genes, including 151 upregulated and 204 downregulated proteins (**Figure 2A**). GO analysis of these proteins indicated their involvement in various biological processes such as cellular metabolic processes, organic substance metabolism, primary metabolic activities, and nitrogen compound metabolism. The proteins were also categorized according to their cellular components and molecular functions (**Figure 2B** and **Supplementary Figure S4**). KEGG pathway analysis revealed that these regulated proteins are associated with several metabolic pathways, including carbohydrate metabolism, amino acid metabolism, lipid metabolism, cofactors and vitamin metabolism, energy metabolism, nucleotide metabolism, glycan biosynthesis, and terpenoid and polyketide metabolism (**Figure 2C**). The subcellular localization of the differentially expressed proteins, including those found in the nucleus, mitochondria, cytoplasm, plasma membranes, and cytoskeletons, is depicted in **Figure 2D**. In the metabolomics aspect, **Figure 2A** summarizes the changes in metabolite levels. The deletion of *IRK5* resulted in distinct metabolic profiles characterized by 394 individual alterations, with 190 metabolites exhibiting increased levels and 204 showing decreased levels. The KEGG enrichment analysis of differentially expressed metabolites is illustrated in **Figure 2E** and **Supplementary Figure S5**. We observed a downregulation in the biosynthesis of aminoacyl-tRNA, biosynthesis of amino acids, metabolic pathways, and steroid biosynthesis (**Supplementary Figure S5**). In contrast, there is an upregulation in the biosynthesis of cofactors (**Supplementary Figure S5**). Overall, the knockout of *IRK5* significantly impacts both protein expression and metabolite levels in *C. neoformans*.

### 3.3 The effect of deleting either *IRK2* or *IRK5* on intracellular ATP levels

**Figure 3:**
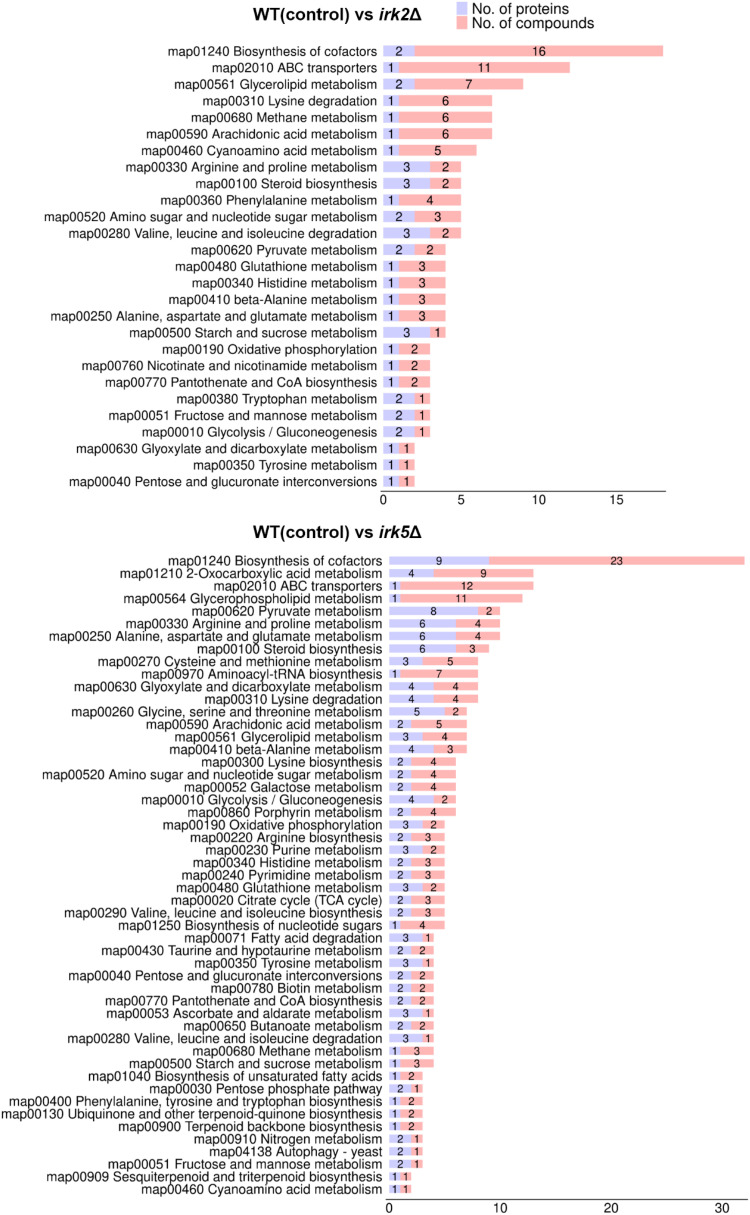
Correlation analyses between proteomic and metabolomic data. Classification of differentially expressed metabolites and proteins by pathway.

**Figure 4:**
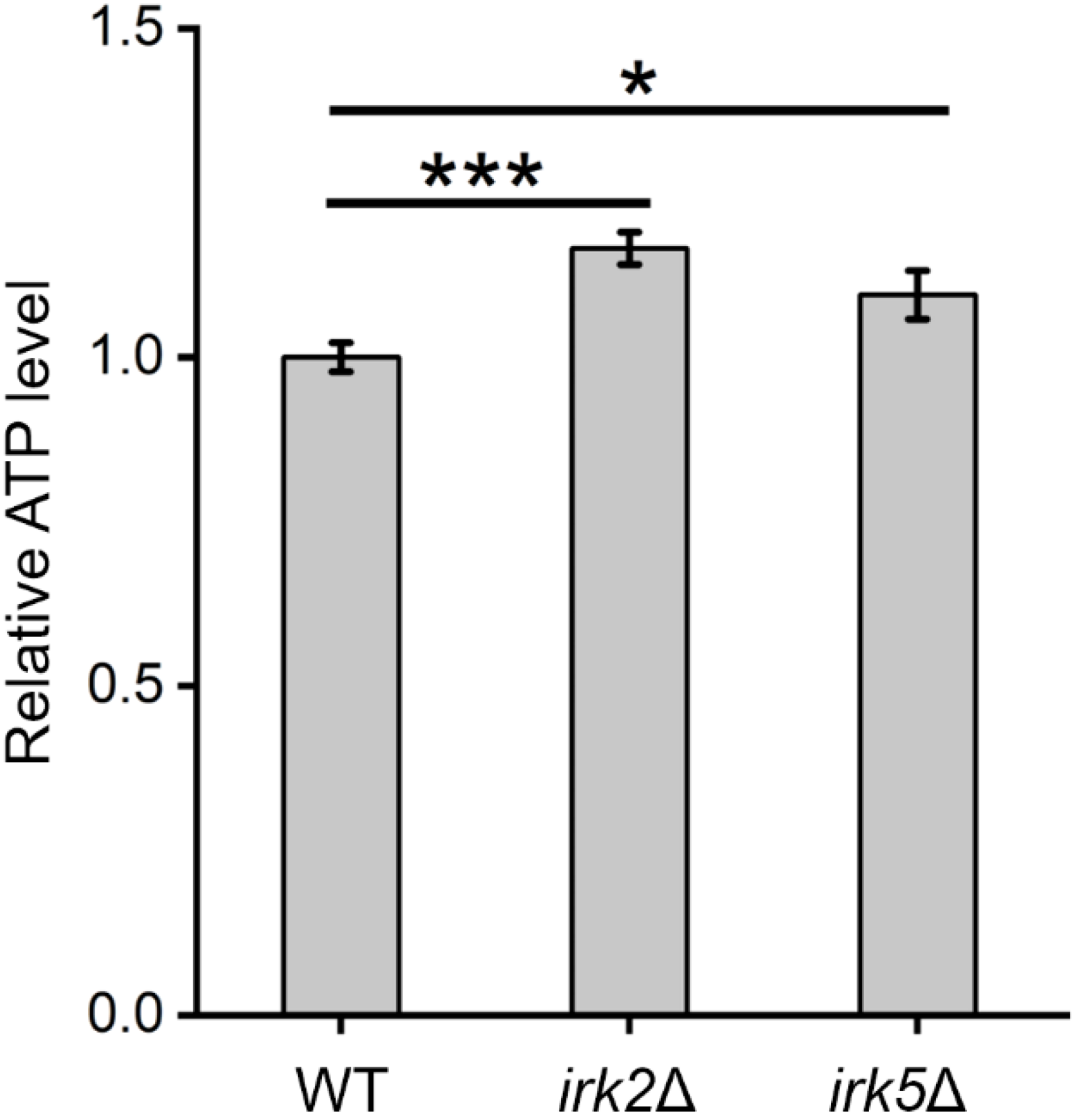
Alterations in intracellular ATP levels were observed following the knockout of *IRK2* or *IRK5*. Significance levels: * (*P*<0.05), *** (*P*<0.001). Luminescence signals were recorded after a 15-minute incubation of fungal cells with BacTiter-Glo™ Reagent.

Both proteomic and metabolomic analyses suggest that the deletion of either *IRK2* or *IRK5* affects the expression of mitochondrial proteins and related metabolites. The subcellular localization of differentially expressed proteins, particularly those associated with mitochondria, is illustrated in **Figure 1D**, **2D**; **Supplementary Tables S3**, **S4**. Notably, the differentially expressed mitochondrial proteins and metabolites involved in oxidative phosphorylation, the TCA cycle or purine metabolism are presented in **Figure 3**. Mitochondria are identified as the main locations for ATP production within cells, where ATP synthesis is intricately associated with oxidative phosphorylation, the TCA cycle, and purine metabolism. The primary pathway for ATP generation is oxidative phosphorylation, while the TCA cycle contributes by producing reducing agents such as NADH and FADH_2_. Furthermore, purine metabolism is crucial for synthesizing ATP, which is vital for numerous cellular activities. Importantly, we observed a significant increase in ATP levels following the knockout of either *IRK2* or *IRK5* (**Figure 4**; **Supplementary Figure S6**). These findings suggest that these kinases may play a significant role in mitochondrial function, underscoring their potential roles in ATP regulation and implications for cellular energetics and virulence.

## 4 Discussion

In this study, we examined the potential roles of the kinases Irk2 and Irk5 in the metabolic regulation of *C. neoformans* through comprehensive proteomic and metabolomic analyses following gene deletions. Our results reveal that the deletion of *IRK2* resulted in significant alterations in the expression of proteins linked to 164 genes, which are involved in various biological processes, particularly metabolic pathways related to carbohydrates, amino acids, lipids, and energy production. Similarly, *IRK5* deletion affected the expression of proteins from 355 genes, impacting comparable metabolic routes. Both mutant strains displayed distinct metabolic profiles, with notable changes in the levels of numerous metabolites. Importantly, we observed a substantial increase in intracellular ATP levels in both *irk2*Δ and *irk5*Δ mutants, indicating a potential regulatory role for these kinases in mitochondrial function and energy metabolism.

Our findings align with previous research highlighting the critical role of protein kinases in the virulence of *C. neoformans*, particularly through their modulation of metabolic pathways that facilitate adaptation and survival within host environments (Hu et al., 2007; Caza and Kronstad, 2019; Choi and Jeong, 2022). Previous studies have identified key kinases that are essential for the pathogenicity of *C. neoformans* (Lee and Hong, 2020). The pronounced modulation of mitochondrial proteins and the consequent elevation in ATP levels observed in our study further underscore the importance of mitochondrial function in the pathogenicity of *C. neoformans*. Mitochondria are integral to energy production, and their role in virulence is increasingly recognized, especially among fungal pathogens that depend on efficient ATP generation for growth and resilience against host defenses. There exists a strong connection between mitochondrial function and virulence in *C. neoformans* (Black et al., 2021; Xue et al., 2023, 2024; Ma et al., 2025). Comparative analyses with other fungal pathogens, such as *Candida albicans* and *Aspergillus fumigatus*, illustrate similar dynamics, where changes in mitochondrial function have profound implications for virulence (Black et al., 2021). In *C. albicans*, the capacity to transition between yeast and hyphal forms—a critical aspect of its pathogenicity—is intricately linked to mitochondrial dynamics and function (Aoki et al., 1989). Likewise, studies on *A. fumigatus* emphasize the necessity of mitochondrial integrity and metabolism for virulence, particularly during invasive infections (Grahl et al., 2012; Neubauer et al., 2015). The increase in ATP levels observed in *C. neoformans* mutants lacking Irk2 or Irk5 suggests that these kinases may act as negative regulators of mitochondrial metabolism, potentially modulating energy production in response to environmental signals during infection. This regulatory mechanism is particularly relevant as *C. neoformans* faces various stressors, including elevated temperatures and oxidative stress, during the host immune response. The differential expression of proteins and metabolites suggests that Irk2 and Irk5 are likely pivotal in fine-tuning the metabolic landscape of *C. neoformans*. In the *irk2*Δ mutant, KEGG enrichment analysis revealed a significant downregulation of several metabolic pathways, particularly those involved in aminoacyl-tRNA and amino acid biosynthesis, which are crucial for fungal virulence (**Supplementary Figure S3**). This downregulation indicates potential suppression of essential metabolic processes. In contrast, the *irk5*Δ mutant also displayed downregulation in similar pathways but revealed an upregulation in cofactor biosynthesis (**Supplementary Figure S5**). This research provides valuable insight into the regulatory mechanisms that govern metabolic adaptation in *C. neoformans*, suggesting potential targets for further study in fungal virulence.

In conclusion, our findings suggest that Irk2 and Irk5 play complex roles in modulating the proteomic and metabolomic profiles of *C. neoformans*, particularly concerning mitochondrial function and ATP production. The observed reductions in protein and metabolite levels in the absence of these kinases may not solely be attributed to their loss; rather, they could result from compensatory mechanisms within the organism. Furthermore, the absence of complemented strains constitutes a significant limitation of this study. Future research should address this gap by implementing alternative strategies to strengthen the conclusions derived from our findings. It is also crucial to note that our experiments were conducted under standard growth conditions, which may not adequately represent the stress-related viability that the study aims to investigate. This limitation emphasizes the necessity for future studies to examine the roles of Irk2 and Irk5 under various stress conditions to comprehensively understand their contributions to the viability of *C. neoformans*.

## Data Availability

The datasets presented in this study can be found in online repositories. The names of the repository/repositories and accession number(s) can be found in the article/**Supplementary Material**.

## Supplementary material

Supplementary Figure 1 Gel images for the PCR confirmation for the mutant *irk2*Δ or *irk5*Δ. 1% TAE agarose gel. The primers used are as follows: Irk2-UP-F: aaaggctccaagtcttgaatattgacat (upstream of *IRK2* gene), Irk2-Down-R: ctcttctattacccctttgctcactt (downstream of *IRK2* gene); Irk5-UP-F: gaagtgcaccatttcaaaatgccg (upstream of *IRK5* gene); Irk5-Down-R: ctgaccctatttccctcagaccg (downstream of *IRK5* gene).

Supplementary Figure 2 Knockout of the *IRK2* resulted in significant changes in protein expression profiles. Proteins exhibiting increased expression levels were analyzed separately from those with decreased expression levels, followed by GO enrichment analysis.

Supplementary Figure 3 Alterations in metabolites were observed following the knockout of the *IRK2*. Metabolites that showed either an increase or decrease in levels were examined independently, followed by KEGG pathway analysis.

Supplementary Figure 4 Deletion of the *IRK5* led to significant alterations in the profiles of protein expression. Proteins displaying either upregulation or downregulation were analyzed separately, followed by GO enrichment analysis.

Supplementary Figure 5 Alterations in metabolites were observed following the deletion of the *IRK5* gene. Metabolites with either an increase or decrease in levels were analyzed separately, followed by KEGG pathway analysis.

Supplementary Figure 6 Changes in intracellular ATP levels were observed in *irk2*Δ or *irk5*Δ mutant. The significance levels were indicated as follows: * (*P* < 0.05) and *** (*P* < 0.001). Luminescence measurements were performed after incubating fungal cells with BacTiter-Glo™ Reagent for 20 minutes.

Supplementary Table 1 List of the primer sequences used for the *irk2*Δ and *irk5*Δ mutants in the WT strain. F: forward primer; R: reverse primer.

Supplementary Table 2 The number of differentially expressed proteins in mutant *irk2*Δ or *irk5*Δ. FC: fold change.

Supplementary Table 3 Differentially expressed proteins localized in the mitochondria were analyzed between the WT control and the *irk2*Δ mutant. For the significantly differentially expressed proteins, the ratio (*irk*2Δ mutant/WT control) exhibited a substantial difference, with a fold change exceeding 1.5 or falling below 0.67, accompanied by a statistically significant p-value of less than 0.05.

Supplementary Table 4 Differentially expressed proteins in mitochondria were compared between the WT control and the *irk5*Δ mutant. Significant differences were identified with a ratio (*irk5*Δ mutant/WT control) showing a fold change greater than 1.5 or less than 0.67, alongside a statistically significant p-value of less than 0.05.
