# Supplementary figures and images for: Evidence for the role of Irk2 and Irk5 in ATP and metabolism regulation in Cryptococcus neoformans
Source: Front Cell Infect Microbiol. 2025 Jun 18;15:1600041. doi: 10.3389/fcimb.2025.1600041 (PMC12214898; doi:10.3389/fcimb.2025.1600041)

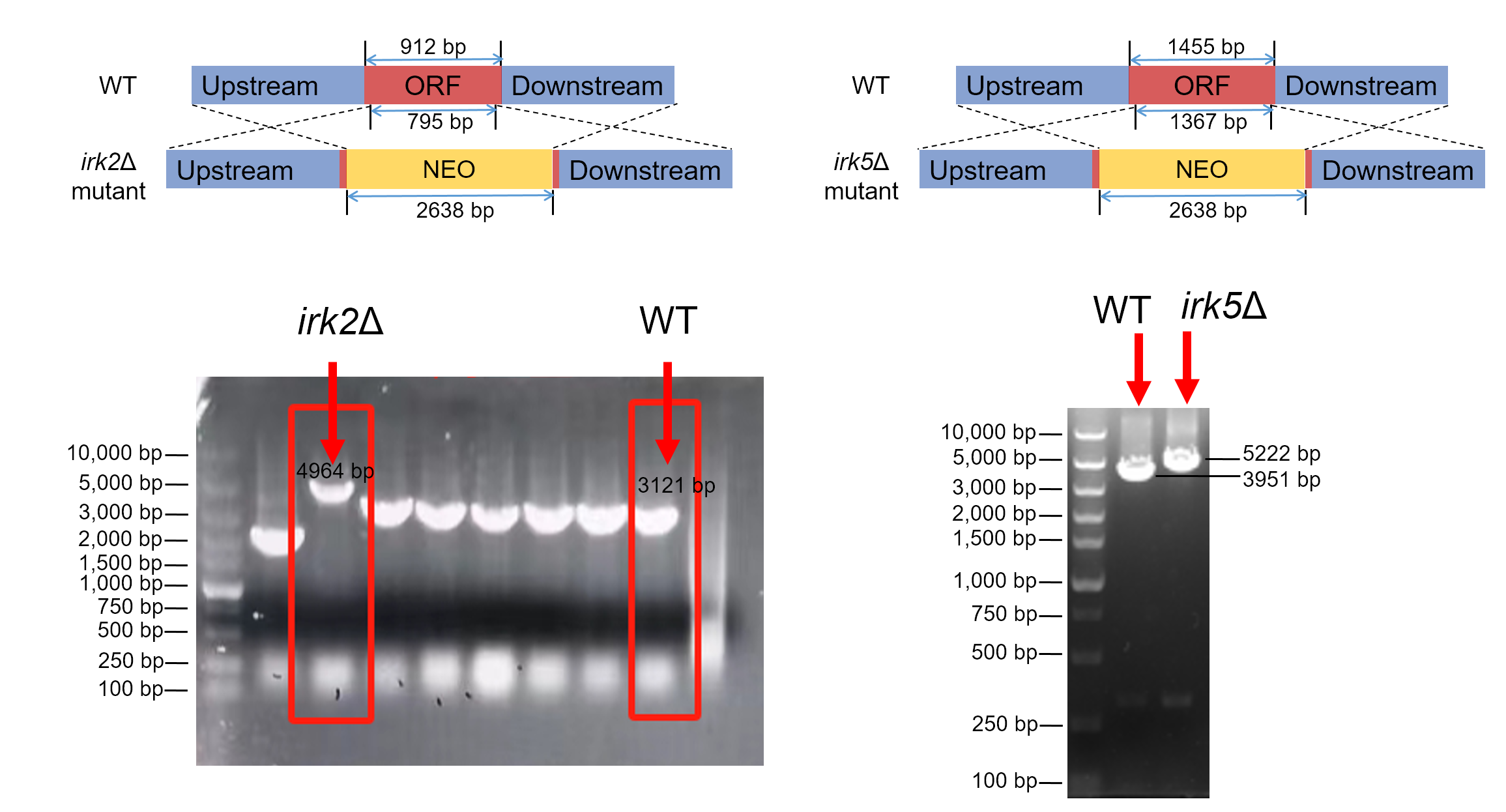

Supplement: Supplementary Figure 1 — Gel images for the PCR confirmation for the mutant irk2Δ or irk5Δ. 1% TAE agarose gel. The primers used are as follows: Irk2-UP-F: aaaggctccaagtcttgaatattgacat (upstream of IRK2 gene), Irk2-Down-R: ctcttctattacccctttgctcactt (downstream of IRK2 gene); Irk5-UP-F: gaagtgcaccatttcaaaatgccg (upstream of IRK5 gene); Irk5-Down-R: ctgaccctatttccctcagaccg (downstream of IRK5 gene). [file Image1.tif]

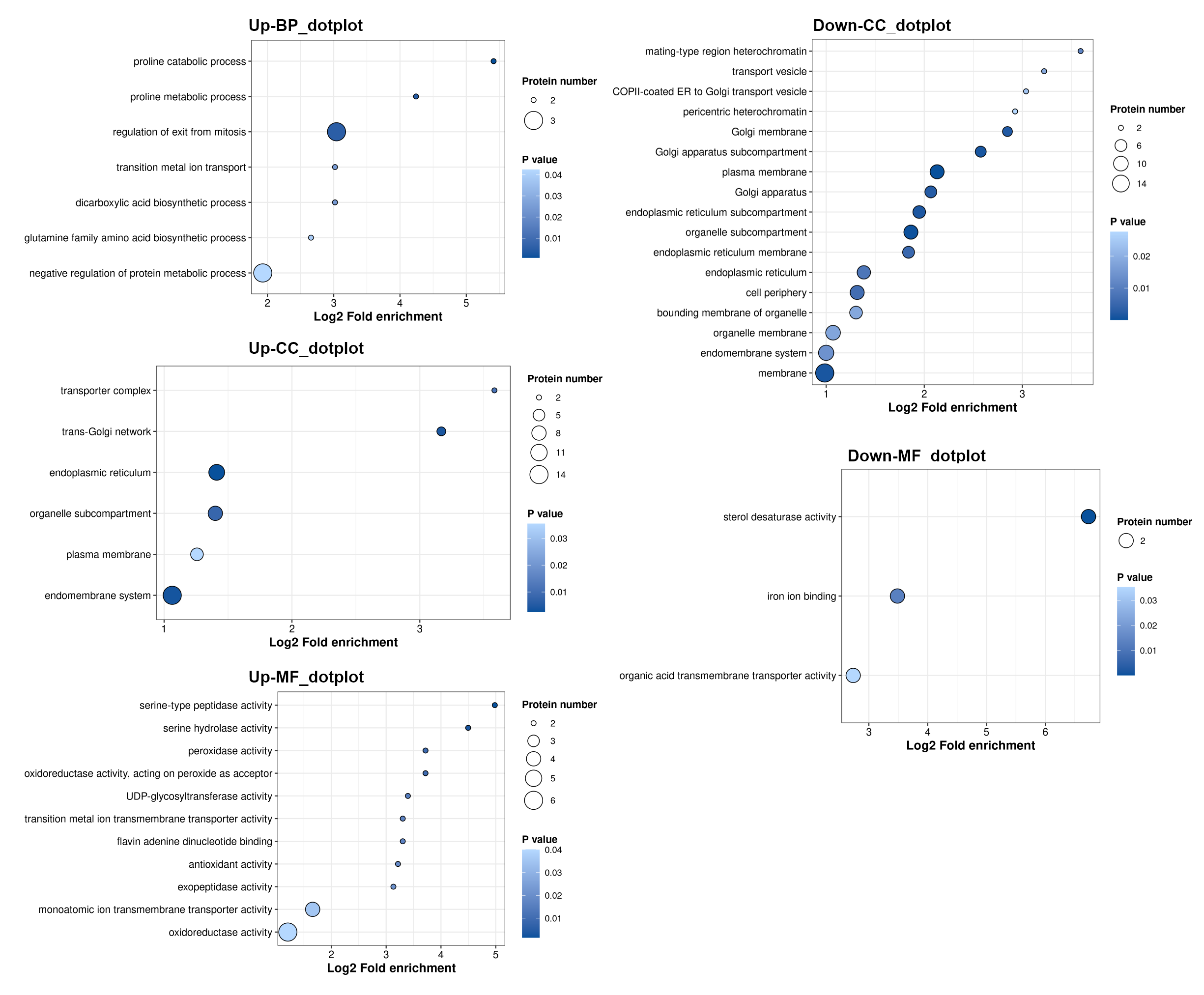

Supplement: Supplementary Figure 2 — Knockout of the IRK2 resulted in significant changes in protein expression profiles. Proteins exhibiting increased expression levels were analyzed separately from those with decreased expression levels, followed by GO enrichment analysis. [file Image2.tif]

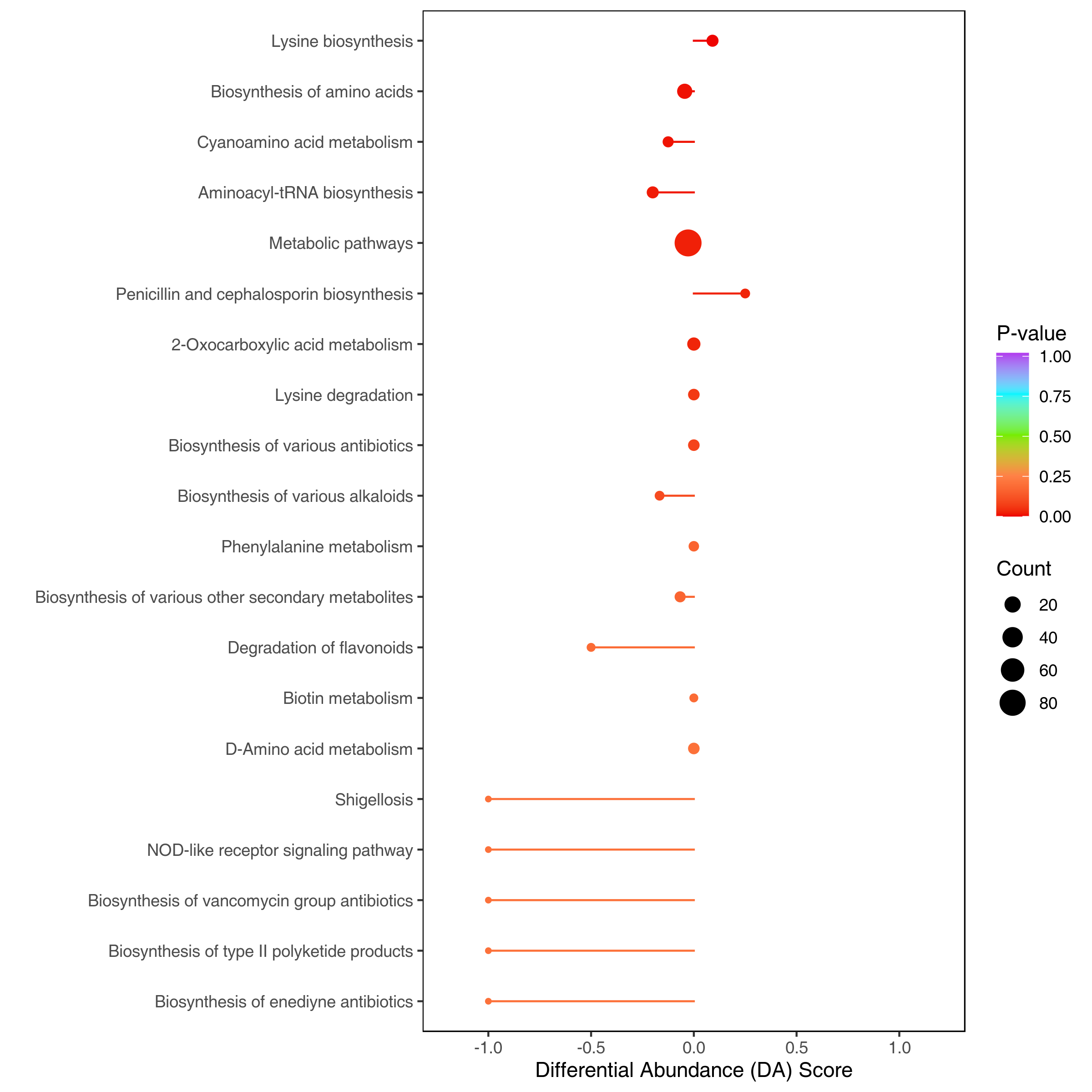

Supplement: Supplementary Figure 3 — Alterations in metabolites were observed following the knockout of the IRK2. Metabolites that showed either an increase or decrease in levels were examined independently, followed by KEGG pathway analysis. [file Image3.tif]

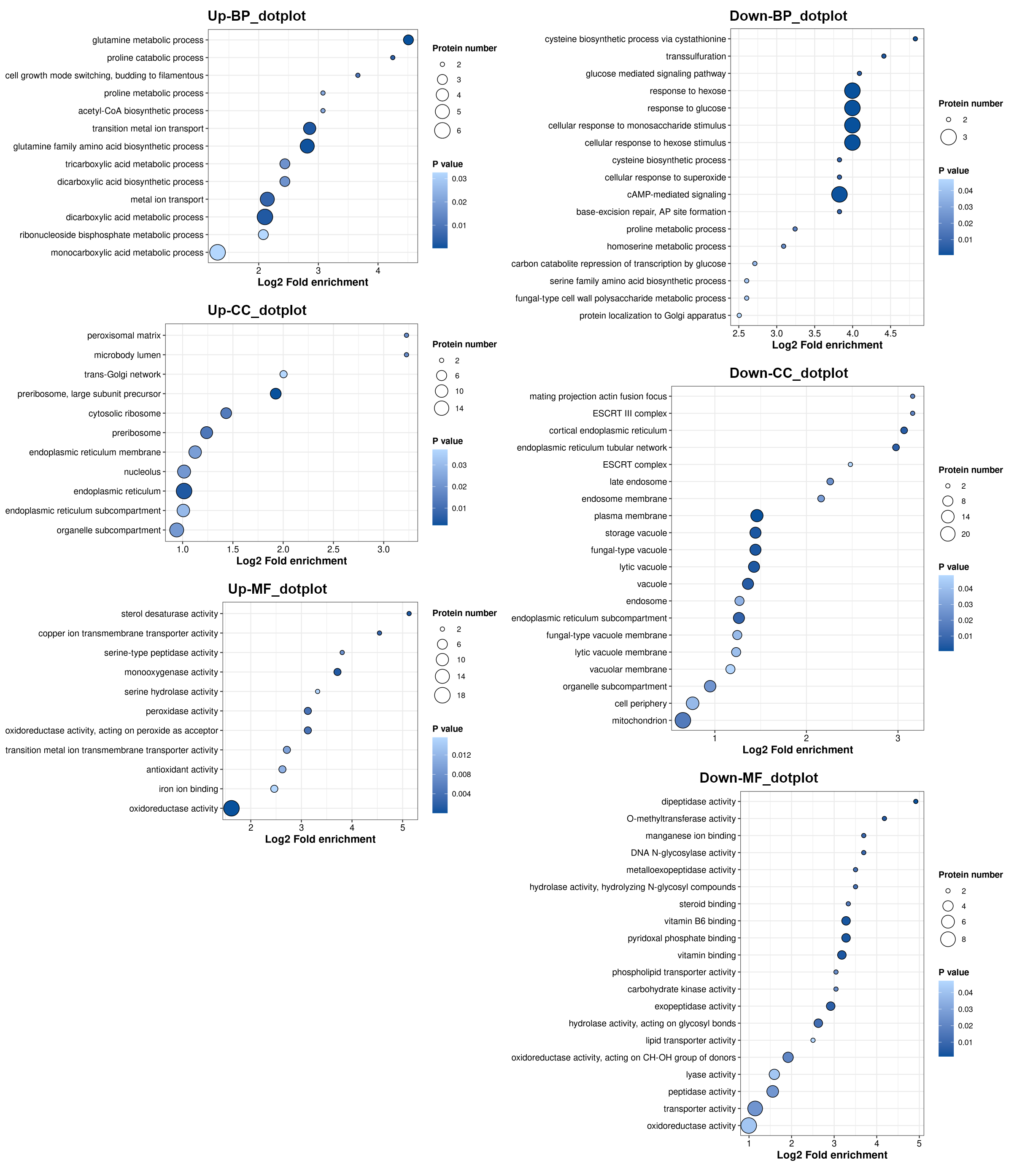

Supplement: Supplementary Figure 4 — Deletion of the IRK5 led to significant alterations in the profiles of protein expression. Proteins displaying either upregulation or downregulation were analyzed separately, followed by GO enrichment analysis. [file Image4.tif]

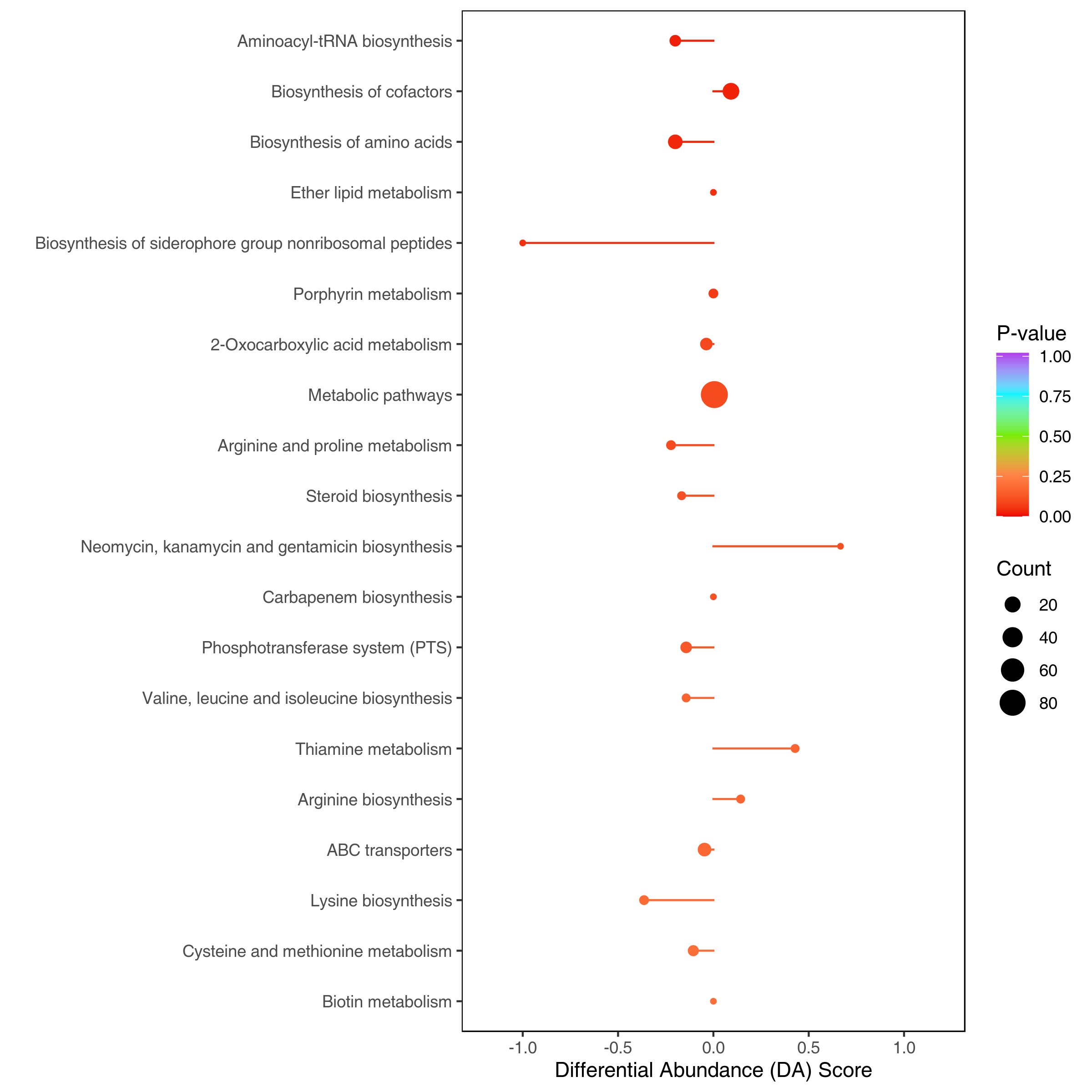

Supplement: Supplementary Figure 5 — Alterations in metabolites were observed following the deletion of the IRK5 gene. Metabolites with either an increase or decrease in levels were analyzed separately, followed by KEGG pathway analysis. [file Image5.tif]

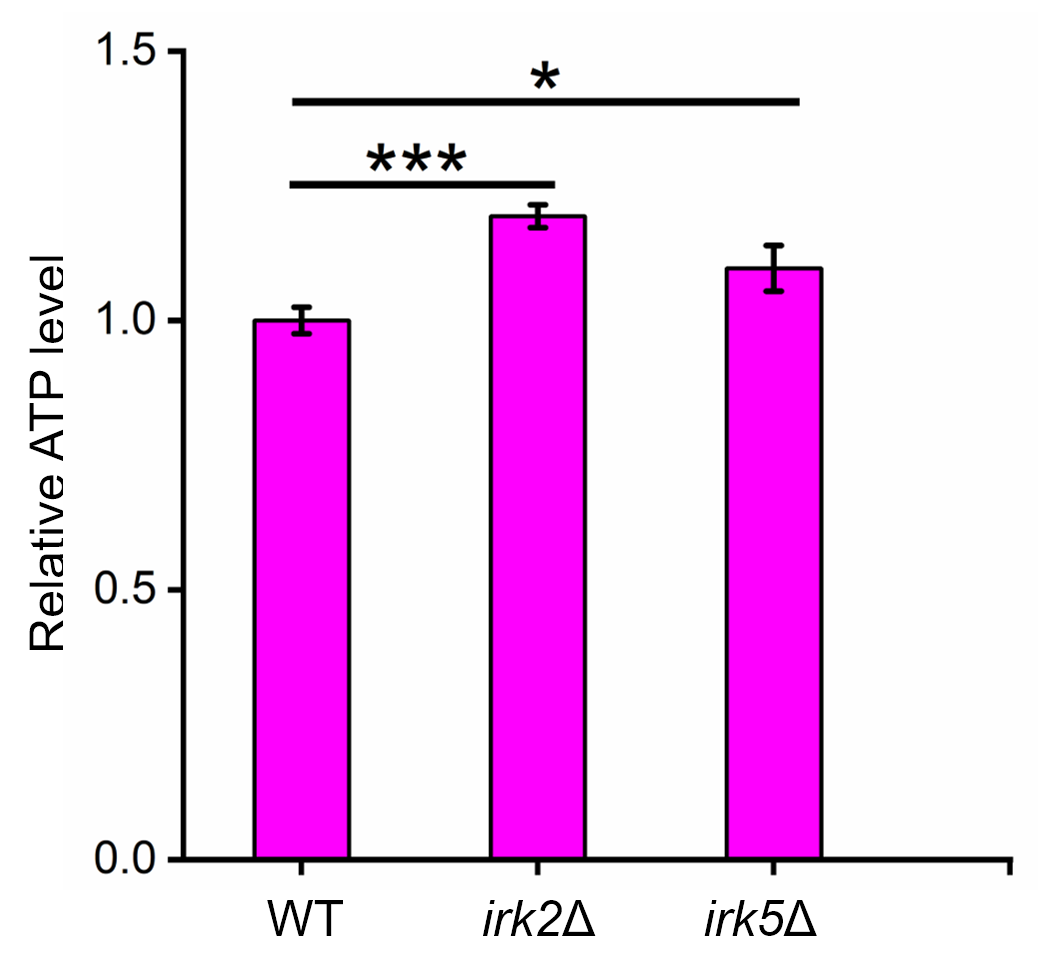

Supplement: Supplementary Figure 6 — Changes in intracellular ATP levels were observed in irk2Δ or irk5Δ mutant. The significance levels were indicated as follows: * (P < 0.05) and *** (P < 0.001). Luminescence measurements were performed after incubating fungal cells with BacTiter-Glo™ Reagent for 20 minutes. [file Image6.tif]
