# Supplementary material for: Evidence for the role of Irk2 and Irk5 in ATP and metabolism regulation in Cryptococcus neoformans
Source: Front Cell Infect Microbiol. 2025 Jun 18;15:1600041. doi: 10.3389/fcimb.2025.1600041 (PMC12214898; doi:10.3389/fcimb.2025.1600041)
Supplement: Supplementary Table 1 — List of the primer sequences used for the irk2Δ and irk5Δ mutants in the WT strain. F: forward primer; R: reverse primer. [file Table1.docx]

**TABLE S1** List of the primer sequences used for the *irk2*Δ and *irk5*Δ mutants in the WT strain. F: forward primer; R: reverse primer.

| **Primers for** ***irk2*Δ mutant in the WT strain** | | |
| --- | --- | --- |
| **Name** | **Sequence 5’-3’** | **Reference** |
| Irk2-UP-F | aaaggctccaagtcttgaatattgacat | This Study |
| Irk2-UP-R | tgagtcgtattacaattcactggccgtcgttttacatgtgagcggctttgaaaatgtttg | This Study |
| neoF | gccggtgttaataataataatgaatctatgatcgggtaaaacgacggccagtgaattgtaatacg | This Study |
| neoR | gaggaaaaaatgataaggtcaacttatactggtatccaggaaacagctatgaccatgattacgc | This Study |
| Irk2-Down-F | atggtcatagctgtttcctgaagttacaaacatggagcgatggg | This Study |
| Irk2-Down-R | ctcttctattacccctttgctcactt | This Study |
| **Primers for *irk5*Δ mutant in the WT strain** | | |
| **Name** | **Sequence 5’-3’** | **Reference** |
| Irk5-UP-F | gaagtgcaccatttcaaaatgccg | This Study |
| Irk5-UP-R | tgagtcgtattacaattcactggccgtcgttttactcgatacgaaattcatacctctggtt | This Study |
| neoF | gccggtgttaataataataatgaatctatgatcgggtaaaacgacggccagtgaattgtaatacg | This Study |
| neoR | gaggaaaaaatgataaggtcaacttatactggtatccaggaaacagctatgaccatgattacgc | This Study |
| Irk5-Down-F | cttggcgtaatcatggtcatagctgtttcctgtatgttgaagcgaaccgtctcc | This Study |
| Irk5-Down-R | ctgaccctatttccctcagaccg | This Study |
