# Supplementary material for: Evidence for the role of Irk2 and Irk5 in ATP and metabolism regulation in Cryptococcus neoformans
Source: Front Cell Infect Microbiol. 2025 Jun 18;15:1600041. doi: 10.3389/fcimb.2025.1600041 (PMC12214898; doi:10.3389/fcimb.2025.1600041)
Supplement: Supplementary Table 2 — The number of differentially expressed proteins in mutant irk2Δ or irk5Δ. FC: fold change. [file Table2.docx]

**TABLE S2** The number of differentially expressed proteins in mutant *irk2*Δ or *irk5*Δ. FC: fold change.

| Knockout | Regulated type | FC >1.2 or <0.83 | FC >1.3 or <0.77 | FC >1.5 or <0.67 | FC >2 or <0.5 |
| --- | --- | --- | --- | --- | --- |
| *IRK2* | Up | 287 | 188 | 92 | 35 |
|  | Down | 256 | 161 | 72 | 26 |
| *IRK5* | Up | 387 | 268 | 151 | 56 |
|  | Down | 484 | 347 | 204 | 49 |
