# Supplementary material for: Evidence for the role of Irk2 and Irk5 in ATP and metabolism regulation in Cryptococcus neoformans
Source: Front Cell Infect Microbiol. 2025 Jun 18;15:1600041. doi: 10.3389/fcimb.2025.1600041 (PMC12214898; doi:10.3389/fcimb.2025.1600041)
Supplement: Supplementary Table 3 — Differentially expressed proteins localized in the mitochondria were analyzed between the WT control and the irk2Δ mutant. For the significantly differentially expressed proteins, the ratio (irk2Δ mutant/WT control) exhibited a substantial difference, with a fold change exceeding 1.5 or falling below 0.67, accompanied by a statistically significant p-value of less than 0.05. [file Table3.docx]

**TABLE S3** Differentially expressed proteins localized in the mitochondria were analyzed between the WT control and the *irk2*Δ mutant. For the significantly differentially expressed proteins, the ratio (*irk*2Δ mutant / WT control) exhibited a substantial difference, with a fold change exceeding 1.5 or falling below 0.67, accompanied by a statistically significant p-value of less than 0.05.

| Protein | Function | WT (control) vs *irk2*Δ mutant |
| --- | --- | --- |
|  |  |  |
| CNAG_02438 | Uncharacterized protein | 1.59 |
| CNAG_07965 | NAD binding dehydrogenase | 1.64 |
| CNAG_08025 | Ribonuclease H1 N-terminal domain-containing protein | 1.55 |
| CNAG_06007 | U3 small nucleolar RNA-associated protein 23 | 1.59 |
| CNAG_04751 | EF-hand domain-containing protein | 2.1 |
| CNAG_06092 | Cyclin | 2.15 |
| CNAG_00873 | Alpha 1,2-mannosyltransferase | 1.63 |
| CNAG_00897 | Glucosidase | 1.68 |
| CNAG_02925 | D-arabinitol 2-dehydrogenase | 1.54 |
| CNAG_02048 | Proline dehydrogenase | 1.54 |
| CNAG_00819 | 40S ribosomal protein S30 | 1.61 |
| CNAG_03240 | alpha-1,2-Mannosidase | 3.58 |
| CCP1 (CNAG_01138) | Cytochrome c peroxidase, mitochondrial | 1.6 |
| CNAG_01261 | Myosin I binding protein | 1.54 |
| CNAG_02288 | Solute carrier family 25 (Mitochondrial citrate transporter), member 1 | 0.5 |
| CNAG_07968 | HIT domain-containing protein | 0.35 |
| CNAG_05573 | Cytochrome c oxidase assembly protein subunit 17 | 0.65 |
| CNAG_04757 | Mitochondrial protein | 0.49 |
| CNAG_06094 | Uncharacterized protein | 0.41 |
| CNAG_01534 | Gram-positive cocci surface proteins LPxTG domain-containing protein | 0.23 |
| CNAG_06297 | NADH dehydrogenase (Ubiquinone) 1 alpha subcomplex 8 | 0.48 |
| CNAG_01588 | Plasma membrane proteolipid 3 | 0.46 |
| CNAG_00997 | Uncharacterized protein | 0.33 |
| CNAG_06644 | C-22 sterol desaturase | 0.62 |
| CNAG_04396 | Uncharacterized protein | 0.53 |
| CNAG_01751 | Uncharacterized protein | 0.63 |
| CNAG_05829 | MIF4G domain-containing protein | 0.63 |
| CNAG_04031 | rRNA methyltransferase 2, mitochondrial | 0.31 |
| CNAG_01385 | Glycosyltransferase family 31 protein | 0.48 |
| CNAG_02284 | 2-oxoisovalerate dehydrogenase subunit alpha | 0.53 |
| CNAG_05859 | Uncharacterized protein | 0.46 |
| CNAG_00233 | Mitochondrial import inner membrane translocase subunit TIM14 | 0.64 |
| CNAG_04830 | Large ribosomal subunit protein bL33m | 0.61 |
| CNAG_07351 | PIN domain-containing protein | 0.57 |
| CNAG_00452 | Isovaleryl-CoA dehydrogenase | 0.56 |
| CNAG_00067 | Protein translocase SEC61 complex gamma subunit | 0.65 |
| CNAG_05081 | Phosphodiesterase | 0.61 |
| CNAG_04088 | Uncharacterized protein | 0.46 |
