# Supplementary material for: Evidence for the role of Irk2 and Irk5 in ATP and metabolism regulation in Cryptococcus neoformans
Source: Front Cell Infect Microbiol. 2025 Jun 18;15:1600041. doi: 10.3389/fcimb.2025.1600041 (PMC12214898; doi:10.3389/fcimb.2025.1600041)
Supplement: Supplementary Table 4 — Differentially expressed proteins in mitochondria were compared between the WT control and the irk5Δ mutant. Significant differences were identified with a ratio (irk5Δ mutant/WT control) showing a fold change greater than 1.5 or less than 0.67, alongside a statistically significant p-value of less than 0.05. [file Table4.docx]

**TABLE S4** Differentially expressed proteins in mitochondria were compared between the WT control and the *irk5*Δ mutant. Significant differences were identified with a ratio (*irk5*Δ mutant / WT control) showing a fold change greater than 1.5 or less than 0.67, alongside a statistically significant p-value of less than 0.05.

| Protein | Function | WT (control) vs *irk5*Δ mutant |
| --- | --- | --- |
| CNAG_03127 | Small subunit ribosomal protein S23 | 2.68 |
| CNAG_03747 | Large subunit ribosomal protein L27Ae | 1.53 |
| CNAG_00149 | NADH dehydrogenase (Ubiquinone) 1 alpha subcomplex 4 SV=1 | 2.29 |
| CNAG_04011 | Large ribosomal subunit protein eL42 | 1.6 |
| CNAG_07802 | Class III aminotransferase | 4.6 |
| CNAG_03165 | Small ribosomal subunit protein mS33 | 1.86 |
| CNAG_00270 | Mitochondrial pyruvate carrier | 1.86 |
| CNAG_06644 | C-22 sterol desaturase | 1.56 |
| CNAG_04751 | EF-hand domain-containing protein | 1.86 |
| CNAG_06517 | Cytoplasmic protein | 1.56 |
| CNAG_01939 | FmHP | 1.67 |
| CNAG_08025 | Ribonuclease H1 N-terminal domain-containing protein | 1.53 |
| CNAG_03267 | Splicing factor 3B subunit 2 | 1.5 |
| AOX1 (CNAG_00162) | Alternative oxidase, mitochondrial | 3.34 |
| CNAG_01542 | Taurine catabolism dioxygenase TauD | 1.9 |
| CNAG_05626 | Thioredoxin domain-containing protein | 1.55 |
| CCP1 (CNAG_01138) | Cytochrome c peroxidase, mitochondrial | 1.62 |
| CNAG_05725 | Ketol-acid reductoisomerase, mitochondrial | 1.58 |
| CNAG_03240 | alpha-1,2-Mannosidase | 3.17 |
| CNAG_05059 | Pyruvate dehydrogenase E1 component subunit beta | 1.5 |
| CNAG_01181 | Large ribosomal subunit protein eL42 | 1.76 |
| CNAG_00457 | Glutamine synthetase | 1.6 |
| CNAG_00854 | C-8 sterol isomerase | 1.9 |
| CNAG_06638 | Malic enzyme | 1.61 |
| CNAG_02048 | Proline dehydrogenase | 1.85 |
| CNAG_06621 | biotin synthase | 2.31 |
| CNAG_07908 | Aconitate hydratase, mitochondrial | 1.84 |
| CNAG_03939 | 5-aminolevulinate synthase | 5.38 |
| CNAG_01721 | hydroxymethylbilane synthase | 2.81 |
| CNAG_06502 | Solute carrier family 25 (Mitochondrial carnitine/acylcarnitine transporter), member 20/29 | 0.65 |
| CNAG_05317 | Phytanoyl-CoA dioxygenase | 0.63 |
| CNAG_05329 | Myo-inositol 2-dehydrogenase | 0.55 |
| CNAG_01102 | Oxidoreductase | 0.65 |
| CNAG_05683 | Uncharacterized protein | 0.56 |
| CNAG_04392 | Sterol-binding protein | 0.66 |
| CNAG_00997 | Uncharacterized protein | 0.42 |
| CNAG_01588 | Plasma membrane proteolipid 3 | 0.47 |
| CNAG_01751 | Uncharacterized protein | 0.5 |
| CNAG_04466 | Selenoprotein O | 0.65 |
| CNAG_02139 | Oxysterol-binding protein | 0.64 |
| CNAG_01495 | Myo-inositol 2-dehydrogenase | 0.6 |
| CNAG_07968 | HIT domain-containing protein | 0.38 |
| CNAG_04288 | Fe-S protein assembly co-chaperone HscB | 0.59 |
| CNAG_06121 | J domain-containing protein | 0.65 |
| CNAG_01534 | Gram-positive cocci surface proteins LPxTG domain-containing protein GN=CNAG_01534 PE=4 SV=1 | 0.19 |
| CNAG_06094 | Uncharacterized protein | 0.27 |
| CNAG_04757 | Mitochondrial protein | 0.66 |
| CNAG_01555 | DDE Tnp4 domain-containing protein | 0.63 |
| CNAG_05421 | Uncharacterized protein | 0.58 |
| CNAG_01991 | Cytochrome c oxidase subunit 4 | 0.51 |
| CNAG_02000 | Short-chain dehydrogenase | 0.55 |
| CNAG_06494 | Cold-induced thioredoxin domain-containing protein | 0.56 |
| CNAG_05859 | Uncharacterized protein | 0.55 |
| CNAG_05390 | Uncharacterized protein | 0.61 |
| CNAG_05829 | MIF4G domain-containing protein | 0.59 |
| CNAG_03213 | UV damage endonuclease UvdE | 0.6 |
| CNAG_02041 | Uncharacterized protein | 0.66 |
| CNAG_03965 | Ribosome recycling factor | 0.61 |
| SOD2 (CNAG_04388) | Superoxide dismutase [Mn], mitochondrial | 0.58 |
| CNAG_00484 | Dihydrolipoamide acetyltransferase component of pyruvate dehydrogenase complex | 0.36 |
| CNAG_04031 | rRNA methyltransferase 2, mitochondrial | 0.32 |
| CNAG_03679 | acylphosphatase | 0.44 |
| CNAG_00735 | aldehyde dehydrogenase (NAD(+)) | 0.21 |
| CNAG_02049 | Proline dehydrogenase | 0.46 |
| CNAG_05429 | histone acetyltransferase | 0.63 |
| CNAG_06051 | Galactokinase | 0.27 |
| CNAG_03927 | Bola-like protein | 0.49 |
| CNAG_04043 | CENP-V/GFA domain-containing protein | 0.56 |
| CNAG_04088 | Uncharacterized protein | 0.42 |
| CNAG_00516 | Peroxin-7 | 0.66 |
| CNAG_00396 | AGC/PKA protein kinase | 0.6 |
| CNAG_05310 | Nipsnap family protein | 0.6 |
| CNAG_03082 | Cupin domain-containing protein | 0.48 |
| CNAG_05173 | DNA-3-methyladenine glycosylase II | 0.64 |
| CNAG_02996 | Flavoprotein oxygenase | 0.61 |
| CNAG_02718 | NAD binding dehydrogenase | 0.52 |
| CNAG_01215 | Cytochrome b mRNA-processing protein 4 | 0.57 |
| CNAG_00247 | Alpha-aminoadipic semialdehyde synthase | 0.12 |
| CNAG_02768 | CENP-V/GFA domain-containing protein | 0.55 |
| CNAG_03869 | Impact N-terminal domain-containing protein | 0.36 |
| CNAG_03874 | Oxidoreductase | 0.55 |
| CNAG_07351 | PIN domain-containing protein | 0.47 |
